# Dental Aesthetics and Self-Esteem of Patients Seeking Orthodontic Treatment

**DOI:** 10.3390/healthcare12161576

**Published:** 2024-08-08

**Authors:** Aufa Dahlia Bahar, Muhammad Syafiee Sagi, Faris Azim Mohd Zuhairi, Wan Nurazreena Wan Hassan

**Affiliations:** 1 Department of Paediatric Dentistry and Orthodontics, Faculty of Dentistry, Universiti Malaya, Kuala Lumpur 50603, Malaysia; aufa.dahlia@um.edu.my; 2 Faculty of Dentistry, Universiti Malaya, Kuala Lumpur 50603, Malaysia; pieesagi@gmail.com (M.S.S.); farisazim@gmail.com (F.A.M.Z.)

**Keywords:** self-esteem, oral health-related quality of life, adolescents, adults, malocclusion

## Abstract

(1) Objective: This study aimed to investigate how patients’ perceptions of their dental aesthetics and their sense of self-esteem are related. (2) Methods: This cross-sectional survey recruited 141 new patients seeking orthodontic treatment. Patients completed a self-administered questionnaire consisting of three parts: the Malaysian Psychosocial Impact of Dental Aesthetics Questionnaire (PIDAQ), Rosenberg Self-esteem Scale (RSES-M), and Aesthetics Component of the Index of Orthodontics Treatment Need (IOTN-AC). Clinical assessment comprised the Dental Health Component of the Index of Orthodontics Treatment Need (IOTN-DHC). Multiple linear regression was performed, with self-esteem as the dependent variable, while the independent variables comprised the domains of PIDAQ; IOTN-AC; IOTN-DHC; and patients’ demographics, such as age, gender, and their level of education. (3) Results: The response rate was 96.5% (*n* = 136 respondents). The multiple linear regression found that, when the other predictors in the model are held constant, Social Impact, Aesthetic Concern, and Dental Self-Confidence are the factors that significantly contributed to explaining the variation in self-esteem, accounting for, 3.9%, 2.3%, and 2.0%, respectively. The entire model explained 23% of the variation in self-esteem. (4) Conclusions: Domains of the psychosocial impact of dental aesthetics act as predictors of self-esteem in orthodontic treatment-seeking patients.

## 1. Introduction

Malocclusion, or problems with dental arrangement, is a global issue. The worldwide prevalence of malocclusion is 56%, regardless of gender [1]. There is substantial evidence regarding the epidemiological significance of malocclusion. Therefore, malocclusion is a significant oral health issue and an economic burden for both the families of affected children and public dental health services.

Orthodontic treatment has anecdotally been perceived to service the demand for addressing problems relating to dental aesthetics. Generally, treatment need for orthodontics is often evaluated by clinicians using normative need indicators [2], which often provide insufficient patient input on the effects of the dental aesthetics on their oral health, emotional, and social factors. The need for orthodontic treatment to manage malocclusion is associated with the oral health-related quality of life in children and adolescents. A systematic review has suggested two potential factors, namely gender and self-esteem, that could modify this relationship [3].

Dental aesthetics, influenced by factors like tooth color, form, and alignment [4], significantly impact adolescents’ psychological well-being and self-confidence [5,6]. Patients who sought orthodontic treatment have been recorded to have a higher psychosocial impact, particularly lower self-confidence [7] than the general population [6] and those who have had orthodontic treatment. Factors that motivate patients to seek orthodontic treatment include wanting to improve their self-image, concerns about aesthetics, and wanting to improve their confidence and to remove their negative thoughts about their teeth [8].

The Psychosocial Impact of Dental Aesthetics Questionnaire (PIDAQ) [9] is a useful tool to assess psychosocial impact related to malocclusion that could support clinical parameters in determining the priority of treatment for patients with malocclusions [10]. Such a validated and reliable instrument could be used as the yardstick to rank patients in terms of priority and expedite treatment to patients with higher psychosocial needs and has the propensity to maintain good oral health during treatment, when the clinical need is comparable [11].

Self-esteem is the belief in our own value and is crucial for mental well-being. Dental aesthetics are potential factors that can impact self-esteem [12]. Poorly aligned teeth or an aesthetically displeasing smile can lead to self-consciousness, reduced confidence, and social anxiety. Peers and social media influence self-esteem. Self-esteem is likely to be lower among those who are unable to adjust to external pressures for personal advancement [13]. For some patients with poor dental aesthetics, an improvement in self-esteem might be anticipated as one of the benefits of orthodontic therapy [14]. However, the evidence for improvement in self-esteem following orthodontic treatment is low [15]. This systematic review also reported that the influence of patients’ gender or age on self-esteem after orthodontic treatment is still not clear.

The Rosenberg Self-Esteem Scale is a recognized tool for self-esteem evaluation [16]. It has been widely used for measuring global self-esteem. The translated scale (RSES-M) has been shown to be a valid and reliable scale for measuring the level of self-esteem among Malaysians [17].

Self-perceptions of smiles and dental aesthetics have been shown to influence the self-esteem of adolescents [18]. However, a previous study found that social factors associated with dental aesthetics were found to be the only significant predictor of self-esteem [19]. The authors related this finding to their culture, wherein other people’s opinion is considered more important than their own. It is unclear whether this can be extended to other populations with varying cultures. Clinical indicators such as malocclusion have been related as causes for low esteem. Adolescents with Class II and III malocclusions reported lower levels of self-esteem scores compared to Class I malocclusion. Thus, there is a need to investigate how malocclusion and other factors, such as oral health-related quality of life, affect self-esteem. The null hypothesis is that there is no significant association between the domains of psychosocial impact of dental aesthetics and the level of self-esteem of patients seeking orthodontic treatment.

Therefore, the objectives of the study were (1) to assess the prevalence, extent, and severity of PIDAQ among patients seeking orthodontic treatment, as well as the level of their self-esteem; (2) to investigate the relationship between the psychosocial impact of dental aesthetics and the self-esteem of patients seeking orthodontic treatment; and (3) to assess the predictive value of the PIDAQ domains on the level of self-esteem. Presumably, the severity of their self-perceived malocclusion and their current self-esteem can be used to identify individuals who are strongly motivated to have orthodontic treatment.

## 2. Materials and Methods

This was a cross-sectional study conducted among patients who were seeking orthodontic treatment at the Faculty of Dentistry, Universiti Malaya. Inclusion criteria were patients who (1) seek orthodontic treatment aged from 12 to 50 years old, (2) have not undergone any orthodontic treatment before, (3) have no language barrier and are able to comprehend English and/or Malay, and (4) have an absence of any craniofacial anomalies. The study was conducted upon ethical approval from the Medical Ethics Committee of Faculty of Dentistry, Universiti Malaya (DF PD2212/0038; 2 April 2022). Respondents were recruited from March 2022 to August 2022.

According to an a priori sample size calculator for multiple regression, at least 118 respondents were needed for an anticipated medium effect size (f^2^ = 0.15), 10 predictors, a power level of 0.80, and a probability of 0.05 [20].

### 2.1. Study Instruments

Questionnaires comprising four sections were self-administered when the patients came for initial consultations at the orthodontic clinic with the postgraduates. The questionnaires were given after consent was given and before the respondents were seen for their orthodontic consultation session. The first section collected demographic information, such as name, age, gender, and education level.

The second section comprised the 22-item Malaysian version of the PIDAQ [21,22] to determine the effects on oral health-related quality of life (OHRQoL) caused by dental arrangement. It assesses three negative domains—Social Impact (SI; 8 items), Psychological Impact (PI; 6 items), and Aesthetic Concern (AC; 2 items)—in addition to a positive domain—Dental Self-Confidence (DSC; 6 items). A 5-point Likert scale, with score 0 meaning “not at all” and score 4 meaning “very strongly”, was used to record the degree of agreement on each item.

The Malaysian version of the Rosenberg Self-Esteem Scale (RSES-M) [23] was used to determine the respondents’ self-esteem score in the third section. It comprises five positive and five negative items. Respondents self-rated the 10 items on a 5-point scale, with 0 signifying “strongly disagree” and 4 signifying “strongly agree”. Respondents with scores below 20 were considered to have low self-esteem [24].

The fourth section used the Aesthetic Component of the Index of Orthodontic Treatment Need (IOTN-AC) to evaluate the respondents’ personal impression of their malocclusion severity. On a scale of one to ten, where one represented the most appealing dental appearance and ten represented the least, respondents were asked to rank their perceptions of their dental aesthetics to determine their self-perceived malocclusion (SPM) [25,26]. In addition, the orthodontic postgraduates, who had been trained on the use of the Dental Health Component of the Index of Orthodontic Treatment Need (IOTN-DHC), clinically assessed for the severity of their malocclusion and thus their need for orthodontic treatment [25]. The postgraduates undergo annual recalibration for the use of the IOTN as part of the curriculum at this institution for screening of all patients.

### 2.2. Data Analysis

Table 1 shows the definitions and score range for the terms used in this study [6,16,25,26]. Overall, only five respondents were removed from analysis due to incomplete demographic information.

For data analysis, the Statistical Package for the Social Sciences (version 27.0; SPSS, Chicago, IL, USA, III) and Winsteps (version 3.80.1; Winsteps, Beaverton, OR, USA) were used; statistical significance was set at *p* < 0.05.

The Shapiro–Wilk tests, Skewness, and Kurtosis distribution were used to determine whether the distribution of the data was normal. The Kruskal–Wallis and Mann–Whitney tests for multiple comparisons were conducted to compare variations in PIDAQ, self-esteem, SPM, and levels of malocclusion severity. For the IOTN-DHC, none of the respondents was rated as grade 1, while respondents with grades 4 and 5 were grouped together for analysis due to a low number of respondents with grade 5. Ordinal scales were converted to interval scales by Rasch analysis [27]. Self-esteem scores between genders were compared using an independent *t*-test, while one-way analysis of variance (ANOVA) was used to compare self-esteem scores between the age groups. The association between malocclusion severity, SPM, PIDAQ, and self-esteem were evaluated using Pearson correlation coefficient. The level of respondents’ self-esteem was tested using multiple linear regression analysis to see if the four PIDAQ domains, as well as age, gender, education level, and the IOTN-DHC and IOTN-AC, significantly predicted a certain level. The model’s multicollinearity was tested using the variance inflation and tolerance factors.

## 3. Results

Table 2 shows the respondents’ profiles. Overall, 136 patients participated in the survey (response rate, 98.6%). There were 50 (36.8%) male and 86 (63.2%) female respondents, with a mean age of 22.4 years (SD: 6.8; range: 12 to 45 years). A majority of respondents (*n* = 71; 52.2%) were between 20 and 29 years old. About a third (*n* = 53; 39.0%) self-perceived themselves as having slight malocclusion, 41 (30.1%) as having moderate malocclusion, and 15 (11.1%) as having severe malocclusion, while 27 (19.9%) did not perceive themselves as having malocclusion. In contrast, when the malocclusion severity level was assessed using IOTN-DHC, as rated by clinicians, only seven (5.1%) respondents were in grade 5 (indicating very great need). Many of the respondents (*n* = 92; 67.6%) were in grade 4 (indicating great need), followed by grade 3 (indicating moderate need) with 24 (17.6%) respondents, and 13 (9.5%) respondents were in grade 2 (indicating little need). For the highest education level among the respondents, 9 (6.6%) have postgraduate qualifications, 60 (44.1%) were degree holders, 17 (12.5%) have a diploma, 47 (34.6%) have up to a secondary-school level of education, and only 3 (2.2%) respondents have a primary level of education.

The psychosocial impact of dental aesthetics of patients seeking orthodontic treatment was highly prevalent, affecting 99.3% of respondents (*n* = 135) (Table 3). The PI was the most prevalent domain, affecting 83% of respondents (*n* = 113), followed by the DSC domain (*n* = 112; 82.4%), the SI domain (*n* = 83; 61%), and the AC domain (*n* = 74; 54.4%). In terms of severity, the mean total PIDAQ score was 52.5, whereas the mean scores for each domain were 16.8 for the DSC, 16.7 for the SI, 14.2 for the PI, and 4.8 for the AC domains.

In terms of the extent of impacts, 14% (*n* = 19) reported significant effects on all domains, 19.9% (*n* = 27) on three domains, 16.9% (*n* = 23) on two domains, 27.2% (*n* = 37) on one domain, and 22.1% (*n* = 30) reported no significant effects on any domains (Table 4). On average, respondents were affected by 1.8 domains.

About 22.1% of respondents had low self-esteem. The mean self-esteem score among all respondents was 24.7 (S.D. = 6.3), with a median score of 25.0 (IQR = 8.75) (Table 5). Respondents between the ages of 40 and 49 years old have the highest mean level of self-esteem (28.5) compared to those who are between 30 and 39 years old and from 20 to 29 years old, who have mean scores of 25.2 and 25.4, respectively (Table 4). Even if the mean of both age groups was almost the same, the self-esteem of respondents between the ages of 20 and 29 years old has a median of 26.0, which is slightly higher than the median of respondents between the ages of 30 and 39 years old (24.0). The age group from 10 to 19 years old had the lowest self-esteem in comparison to all the age groups, with a mean score of 23.1. However, the one-way ANOVA of the person measure values found no significant differences in the self-esteem between patients of the different age groups (F (132, 3) = 1.793; *p* = 0.152). The self-esteem scores between male (mean = 24.0, median = 25.0) and female respondents (mean = 25.1, median = 24.5) were almost similar (Table 5). The independent *t*-test showed no differences in the self-esteem scores between genders (*p* > 0.05). 

**Figure 1:**
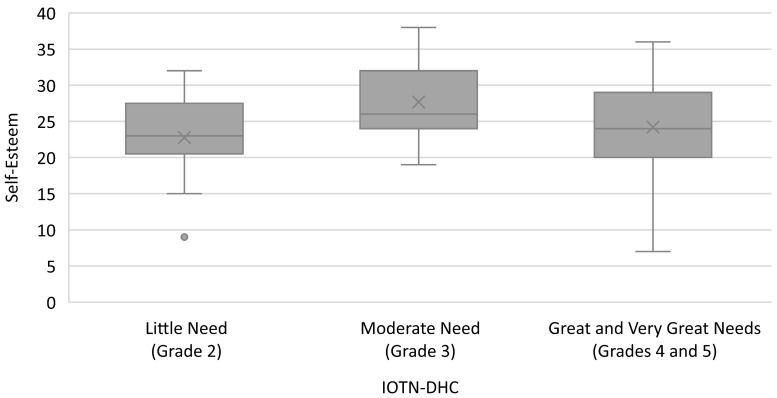
Comparison between self-esteem and malocclusion-severity levels, as measured by the Dental Health Component of the Index of Orthodontic Treatment Need (IOTN-DHC).

Figure 1 shows that individuals with moderate need (grade of 3) for treatment have a greater self-esteem level than respondents with little need (grade of 2) and severe need (grades of 4 and 5). Self-esteem did not significantly differ between PIDAQ domains and self-rated IOTN-AC assessments of SPM. The Kruskal–Wallis test found that the self-esteem of patients seeking orthodontic treatment were different in regard to the different levels of severity of malocclusion, as measured using IOTN-DHC (*p* < 0.05). A post hoc analysis by Mann–Whitney tests showed significant differences in self-esteem scores between those with little need and moderate need (*p* = 0.032) and between moderate need and severe need (*p* = 0.029) for orthodontic treatment.

According to Pearson correlation coefficient (Table 6), self-esteem had a moderately negative correlation with SI (r= −0.425, *p* < 0.01), a weakly negative correlation with PI (r = −0.321, *p* < 0.01), a weakly negative correlation with reversed DSC (r = −0.298, *p* < 0.01) domains, a weakly negative correlation with education level (r = 0.217, *p* < 0.01), and a weakly negative correlation with age (r = 0.185, *p* < 0.05). On the other hand, neither gender, professionally rated IOTN-DHC, nor self-reported IOTN-AC was significantly correlated with self-esteem.

According to the regression analysis (Table 7), the nine predictors explained 23% of the variance (R^2^ = 0.28, F (9, 126) = 5.483, *p* < 0.001). The SI (β = −0.386, *p* < 0.001), AC (β = 0.228, *p* < 0.05), and reversed DSC (β = −0.195, *p* < 0.05) domains were found to significantly predict self-esteem. In this model, other factors, such as PI, self-rated IOTN-AC, professional-rated IOTN-DHC, age, gender, and education level, did not significantly predict self-esteem.

## 4. Discussion

### 4.1. Prevalence, Extent, and Severity of PIDAQ among Patients Seeking Orthodontic Treatment

This study found that PIDAQ prevalence and severity were higher than in previous epidemiological studies on young adults [28] and schoolchildren [6]. Our respondents’ mean PIDAQ score was similar to that in a longitudinal study assessing the PIDAQ of patients prior to fixed appliances therapy [7]. Another study assessing PIDAQ after one year of orthodontic treatment also showed similar pre-treatment scores as this current study, with a significant reduction in the mean values after one year in treatment [29]. This proves that patients who seek orthodontic treatment have a strong psychosocial impact of their malocclusion and, hence, a worse quality of life compared to individuals who perceive themselves as having no or minimal malocclusion.

The respondents of this study experienced similar impacts on both DSC and PI domains. Other studies found PI to be the most prevalent, but in terms of the extent of impact, we had similar results [6,28]. This suggests that adolescents and adults seeking orthodontic treatment are more psychologically impacted by malocclusion.

### 4.2. Self-Esteem and PIDAQ

Our study found an association between self-esteem and the PIDAQ domains, with SI having a moderate negative association with self-esteem (r = −0.425; *p* < 0.05), followed by PI (r = −0.321; *p* < 0.05). On the other hand, DSC and AC had a weak association with self-esteem, as found by a weak negative association between the reverse-scored DSC with self-esteem (r = −0.298; *p* < 0.05) and between AC with self-esteem (r = −0.159; *p* < 0.05). Our findings were similar to studies on the Croatian and Chinese populations [19,30]. A study among university students also reported that individuals experiencing substantial psychosocial effects due to dental aesthetics are likely to exhibit lower levels of self-esteem [31]. This suggested that, regardless of cultural differences in the Western or Eastern community, negative social behaviors and dissatisfaction over one’s own dental aesthetics might influence the subjective impressions of an individual, such as self-esteem.

SI, AC, and reverse scored DSC domains were significant predictors of self-esteem in our study based on the regression model analysis. People with higher self-esteem have lower results in the SI domain, which explains that they care less about their dental appearance in social settings. However, people with higher self-esteem have higher results in the AC domain, which means that they are more aware of their physical appearance. Additionally, people with higher self-esteem have lower results in the reversed DSC domain, depicting that satisfaction with dental appearance has a positive effect on self-esteem [19]. Although the PI domain and self-esteem are correlated, the domain did not significantly predict self-esteem in our study.

### 4.3. Self-Esteem and the Degree of Malocclusion

Our study found no correlation between malocclusion severity and self-esteem, with respondents with little need of treatment having the lowest self-esteem and those with moderate treatment need having the highest self-esteem levels. Research suggests that self-esteem is a complex issue that is not solely dependent on malocclusion [32,33,34,35], and contentment with one’s appearance can boost self-esteem [36].

Poor dental aesthetics can increase patients’ levels of anxiety, particularly those with high tendencies toward perfectionism. The constant concern over the teeth can increase their levels of depression [37]. Luo et al. (2021) suggested incorporating psychological intervention as part of patient management to properly improve the patients’ aesthetic orientation, reduce their expectations, and increase their satisfaction.

Being female and having poor dental aesthetics are factors for poor self-esteem [38]. On the other hand, orthodontic treatment is an important factor that can improve the self-esteem of adolescents [38]. A study found that orthodontic treatment improves self-esteem as early as 3 months into treatment and continues to increase after a year’s follow-up [39]. Other studies also similarly found patients having higher self-esteem levels post-treatment compared to pre-treatment and during treatment [40,41].

In another study, it was found that global self-esteem remains stable throughout orthodontic treatment. This study found an interaction effect between time into treatment with age and gender for certain self-esteem subscales. For the scholastic competence subscale, the values reduce in girls, while in boys, the values increase during treatment until the end of treatment. Adolescents who started orthodontic treatment at a younger age have been found to have increased levels in the physical-appearance and global self-worth subscales after one year of treatment, whereas older adolescents showed a trend toward decreased self-esteem; however, at the end of treatment, both groups had similar levels of self-esteem [42].

These findings suggest that self-esteem is influenced by various elements, such as body image, facial appearance, levels of anxiety and depression, and social acceptance.

### 4.4. Self-Esteem of Various Age Groups, Gender, and Education Levels

According to the findings of our research, there is a correlation between increasing age and increased level of total self-esteem, supporting the finding of a previous longitudinal study [43]. People generally become more accepting of who they are as they get older [44]. Nonetheless, it should be kept in mind that the trajectory of self-esteem change may differ across cultures [45] and the environment that one is exposed to.

The current study found no significant difference in self-esteem between genders, despite previous research showing lower self-esteem in adolescent girls [46,47,48,49] and that malocclusion had a greater impact on girls’ self-esteem [47]. However, male self-esteem rises, and female self-esteem falls from early adolescence to early adulthood [50]. Another local study found that males and females in urban areas had similar PIDAQ scores, while males in sub-urban and rural areas had lower PIDAQ scores than their female counterparts [28]. This finding explained why our current study has no significant differences between the genders, as our respondents were from urban areas of Kuala Lumpur and Selangor.

Our research indicates that an individual’s self-esteem increases with his or her education level, consistent with a previous study indicating higher self-esteem among those with higher education [51].

### 4.5. Study Limitations

The study faced limitations due to short data collection time and five missing data samples. Despite this, the final sample size was sufficient for power. A majority of the respondents were adults between 20 and 29 years old (52.2%), followed by the combination of adolescents and young adults of between 10 and 19 years old (33.8%). Despite the wide age range of the respondents, their self-esteem scores were generally stable, with no significant differences across the age groups. Therefore, the results of this study could be applied to a wide age group.

## 5. Conclusions

The prevalence of psychosocial impacts of dental aesthetic amongst patients seeking orthodontic treatment was 99.3%, with 22.1% showing low self-esteem. The significant predictors of self-esteem of orthodontic treatment-seeking patients were found to be Social well-being, Aesthetic Concern, and Dental Self-Confidence.

## Figures and Tables

**Table 1:** Definitions and score range of the terms.

Term	Definition	Score Range
Psychosocial Impact of Dental Aesthetics Questionnaire (PIDAQ) and PIDAQ domains		
Significant impact item	Item with responses of strongly (score 3) and very strongly (score 4) for the negative SI, PI, and AC domains, and not at all (score 0) and a little (score 1) for the DSC domain.	
Prevalence	The percentage of respondents who reported having any significant impact item for each domain and overall PIDAQ.	0 to 100%
Extent	(1) The number of domains with at least one significant impact item.	0 to 4
	(2) The percentage of respondents who reported having a significant impact item on one or more PIDAQ domains.	0 to 100%
Severity *	(1) The severity for total PIDAQ is the total response codes for each item in the SI, PI, and AC domains and reverse scores codes for the DSC domain.	PIDAQ: 0 to 88
	(2) The severity for each PIDAQ domain is the total response codes for each item in the domain. For the DSC domain, reverse scores were used.	DSC: 0 to 24
		PI: 0 to 24
		SI: 0 to 32
		AC: 0 to 8
Rosenberg Self-Esteem Scale, Malaysian Version (RSES-M)		
Scale ^#^	Overall score is calculated by summing the scores of the five positive items and the inverted scores of the five negative items of the RSES-M scale.	0 to 40
Aesthetic Component of the Index of Orthodontic Treatment Need (IOTN-AC)		
No SPM	Respondents who rated their Self-Perceived Malocclusion (SPM) as 1 to 2 on the IOTN-AC scale.	
Slight SPM	Respondents who rated themselves as 3 to 4 on the IOTN-AC scale.	
Moderate SPM	Respondents who rated themselves as 5 to 7 on the IOTN-AC scale.	
Severe SPM	Respondents who rated themselves as 8 to 10 on the IOTN-AC scale.	
Dental Health Component of the Index of Orthodontic Treatment Need (IOTN-DHC)		
No need	Respondents who were rated as 1 on the IOTN-DHC scale.	
Little need	Respondents who were rated as 2 on the IOTN-DHC scale.	
Moderate need	Respondents who were rated as 3 on the IOTN-DHC scale.	
Great need	Respondents who were rated as 4 on the IOTN-DHC scale.	
Very great need	Respondents who were rated as 5 on the IOTN-DHC scale.	

Psychosocial Impact of Dental Aesthetics (PIDAQ), Dental Self-Confidence (DSC), Psychosocial Impact (PI), Social Impact (SI), and Aesthetic Concern (AC), * Higher scores indicate more severe psychosocial impacts, ^#^ A higher score corresponds to a greater self-esteem’s scale.

**Table 2:** Respondents’ profile, based on gender and age.

Index of Treatment Need	Gender *n* (%)		Age Range (Years) *n* (%)				Total *n* (%)
	Male	Female	10–19 y	20–29 y	30–39 y	40–49 y	136 (100%)
Aesthetic Component (Respondent Rated)							
No SPM	9 (6.6)	18 (13.2)	12 (8.8)	14 (10.3)	1 (0.7)	0	
Mild SPM	19 (14.0)	34 (25.0)	18 (13.2)	29 (21.3)	5 (3.7)	1 (0.7)	
Moderate SPM	17 (12.5)	24 (17.6)	9 (6.6)	23 (16.9)	7 (5.1)	2 (1.5)	
Severe SPM	5 (3.7)	10 (7.4)	7 (5.1)	5 (3.7)	2 (1.5)	1 (0.7)	
Dental Health Component (Clinician Rated)							
Little need	2 (1.5)	11 (8.1)	5 (3.7)	5 (3.7)	3 (2.2)	0	
Moderate need	7 (5.1)	17 (12.5)	5 (3.7)	16 (11.8)	2 (1.5)	1 (0.7)	
Great need	37 (27.2)	55 (40.4)	33 (24.3)	48 (35.3)	9 (6.6)	2 (1.5)	
Very great need	4 (2.9)	3 (2.2)	3 (2.2)	2 (1.5)	1 (0.7)	1 (0.7)	

Self-perceived malocclusion (SPM).

**Table 3:** Prevalence and severity scores of PIDAQ among patients seeking orthodontic treatment.

	Prevalence		Severity	
	N	%	Mean	S.D.
Total PIDAQ	136	99.3	52.5	14.9
DSC	112	82.4	16.8	4.0
PI	113	83.0	14.2	5.0
SI	83	61.0	16.7	7.2
AC	74	54.4	4.8	1.8

Psychosocial Impact of Dental Aesthetics (PIDAQ), Dental Self-Confidence (DSC), Psychosocial Impact (PI), Social Impact (SI), and Aesthetic Concern (AC).

**Table 4:** Extent of PIDAQ among patients seeking orthodontic treatment.

Frequency, N (%)					Descriptives			
0	1	2	3	4	Mean	S.D.	Range	Quartiles
30 (22.1)	37 (27.2)	23 (16.9)	27 (19.9)	19 (14.0)	1.8	1.4	0–4	(1, 2, 3)

**Table 5:** Self-esteem scores for different age groups and genders (*n* = 136).

Age Range	Male (*n* = 50)					Female (*n* = 86)					Independent *t*-Test	Overall (*n* = 136)				
(Years)	*n* (%)	Mean	S.D.	Median	Interquartile Range	*n* (%)	Mean	S.D.	Median	Interquartile Range	*p*-Value	*n* (%)	Mean	S.D.	Median	Interquartile Range
10–19	19 (38.0)	22.7	6.7	21.0	10.0	27 (31.4)	23.4	5.9	24.0	8.0	0.894	46 (33.8)	23.1	6.2	23.0	9.0
20–29	26 (52.0)	25.1	7.9	26.5	9.0	45 (52.3)	25.5	5.4	25.0	8.0	0.918	71 (52.2)	25.4	6.4	26.0	8.0
30–39	5 (10.0)	23.4	5.1	23	10.0	10 (11.6)	26.1	6.2	25.5	11.0	0.380	15 (11.0)	25.2	5.8	24.0	9.0
40–49	0	-	-	-	-	4 (4.7)	28.5	5.4	28.5	11.0	-	4 (3.0)	28.5	5.4	28.5	10.5
All	50 (100%)	24.0	7.2	25.0	11.0	86 (100%)	25.1	5.7	24.5	7.25	0.595	136 (100)	24.7	6.3	25.0	9.0

**Table 6:** Pearson correlations between domains of PIDAQ with age, gender, and education level.

		DSC ^§^	SI	PI	AC	IOTN-AC	IOTN-DHC	Age	Gender	Education Level
Self-esteem	r	−0.298	−0.425	−0.321	−0.159	−0.13	−0.06	0.185 *	0.046	0.217
	*p*	0.000 *	0.000 *	0.000 *	0.032 *	0.065	0.246	0.015	0.297	0.006 *
DSC ^§^	r	1	0.357	0.266	0.456	0.427	0.092	−0.116	−0.073	−0.142
	*p*		0.000 *	0.001 *	0.000 *	0.000 *	0.142	0.09	0.198	0.050 *
SI	r		1	0.827	0.579	0.189	−0.054	0.036	0.034	−0.026
	*p*			0.000 *	0.000 *	0.014 *	0.266	0.34	0.346	0.381
PI	r			1	0.598	0.281	−0.11	0.208 *	0.126	0.068
	*p*				0.000 *	0.000 *	0.100	0.008	0.073	0.215
AC	r				1	0.256	−0.022	0.082	0.010	−0.063
	*p*					0.001 *	0.399	0.17	0.453	0.235
IOTN-AC	r					1	0.168	0.133	−0.03	−0.062
	*p*						0.025 *	0.062	0.363	0.237
IOTN-DHC	r						1	−0.009	−0.188	0.028
	*p*							0.458	0.014 *	0.371
Age	r							1	0.144	0.558
	*p*								0.048 *	0.000 *
Gender	r								1	0.133
	*p*									0.061

* Correlation is significant at the 0.05 level. DSC ^§^ = reversed DSC.

**Table 7:** Multiple linear regression for prediction of self-esteem.

**Model**			**Unstandardized** **Coefficients**		**Standardized Coefficients**			**Correlations**		
			**B**	**Std. Error**	**Beta**	**t**	***p***	**Zero Order**	**Partial**	**Part**
1	(Constant)		0.526	0.553		0.951	0.343			
	AC		0.088	0.040	0.228	2.203	0.029 *	−0.159	0.193	0.166
	SI		−0.254	0.096	−0.386	−2.648	0.009 *	−0.425	−0.230	−0.200
	PI		−0.079	0.094	−0.130	−0.843	0.401	−0.321	−0.075	−0.064
	Reversed DSC		−0.103	0.050	−0.195	−2.036	0.044 *	−0.298	−0.178	−0.154
	IOTN-AC		0.017	0.110	0.014	0.150	0.881	−0.130	0.013	0.011
	IOTN-DHC		−0.118	0.124	−0.075	−0.948	0.345	−0.060	−0.084	−0.072
	Age		0.016	0.016	0.097	0.997	0.321	0.185	0.088	0.075
	Gender		0.026	0.182	0.011	0.144	0.886	0.046	0.013	0.011
	Education Level		0.159	0.099	0.150	1.609	0.110	0.217	0.142	0.121
**Model Summary** **^b^**							**ANOVA**			
Model		R	R square	Adjusted R square		Std. error of the estimate	F	Df1	Df2	Sig.
1		0.530 ^a^	0.281	0.230		0.98460	5.483	9	126	0.000

^a^ Predictors: (constant), education level, SI, IOTN-DHC(C), gender, IOTN-AC(P), DSC, age, AC, and PI. ^b^ Dependent variable: self-esteem. * *p* < 0.05.

## Data Availability

The data presented in this study are openly available in UM Research Repository at http://eprints.um.edu.my/id/eprint/45074 (accessed on 26 June 2024).
